# Supplementary material for: The relationship between family history of cancer and cancer attitudes & beliefs within the Community Initiative Towards Improving Equity and Health Status (CITIES) cohort
Source: PLoS One. 2023 Jun 27;18(6):e0287629. doi: 10.1371/journal.pone.0287629 (PMC10298770; doi:10.1371/journal.pone.0287629)
Supplement: Attachment — Submitted filename: ResponsetoReviewers_CITIES.docx [file pone.0287629.s001.docx]

Thank you for your thoughtful review, comments, and suggestions. We believe we have now addressed these concerns and have detailed them below.

Academic Editor comments:

- Please remove Wald from 95 % CI in tables titles

*RESPONSE: Wald has been removed from all table titles.*

- References in the conclusion section may be moved to the discussion part with focus on conclusion only.

*RESPONSE: References in the conclusion section have been removed and now are addressed in the discussion section.*

- 203 line (Backward Model Selection) remove it and add it either in the footnote or text

*RESPONSE: Thank you, (Backward Model Selection) was removed and added to the footnote for all relevant tables.*

- replace N with small n (N=603) in all the places

*RESPONSE: All N’s have been replaced with small n’s.*

- correct the spacing of all table titles

*RESPONSE: Thank you, we have changed the spacing to single spacing for all table titles, if there are other concerns, we are more than happy to edit with more clarification from the reviewer.*

- there is difference and bias introduced while using multiple data collection methods like survey through phone calls, in-person interviews, and web

*RESPONSE: Thank you, we agree with this comment. The strengths and limitations section has been updated to reflect this.*

Additional requirements:

1. Please ensure that your manuscript meets PLOS ONE's style requirements, including those for file naming.

*RESPONSE: Thank you. This has been addressed.*

2. Thank you for stating in your Funding Statement:

“This project was supported by The Ohio State University Comprehensive Cancer Center using Pelotonia funds, and a supplement to the NCI grant (P30CA016058), The Ohio State University Center for Clinical and Translational Science CTSA grant UL1TR002733, and supported by the Recruitment, Intervention and Survey Shared Resource (RISSR) at The Ohio State University Comprehensive Cancer Center (P30CA016058). This work was supported by the Samuel J. Roessler Memorial Scholarship through The Ohio State University College of Medicine’s Medical Student Research Program Scholarship (To LL) and the National Cancer Institute (F99CA25374501 to XZ). The funders had no role in study design, data collection and analysis, decision to publish, or reparation of the manuscript.”

Please provide an amended statement that declares *all* the funding or sources of support (whether external or internal to your organization) received during this study, as detailed online in our guide for authors at [http://journals.plos.org/plosone/s/submit-now.](https://urldefense.com/v3/__http:/journals.plos.org/plosone/s/submit-now.__;!!AU3bcTlGKuA!AiTJulXq0ltDHMT5CiHXiTIiUjshdPDrUjs1EAgZ_RHBTTRSeScTE06vQ3YRKAtNGSAym27V40gdxB2w$)  Please also include the statement “There was no additional external funding received for this study.” in your updated Funding Statement.

Please include your amended Funding Statement within your cover letter. We will change the online submission form on your behalf.

*RESPONSE: the Financial Statement has now been updated and is included in the cover letter.*

3. Please include your full ethics statement in the ‘Methods’ section of your manuscript file. In your statement, please include the full name of the IRB or ethics committee who approved or waived your study, as well as whether or not you obtained informed written or verbal consent. If consent was waived for your study, please include this information in your statement as well.

*RESPONSE: Thank you. This has been addressed.*

4. Please review your reference list to ensure that it is complete and correct. If you have cited papers that have been retracted, please include the rationale for doing so in the manuscript text, or remove these references and replace them with relevant current references. Any changes to the reference list should be mentioned in the rebuttal letter that accompanies your revised manuscript. If you need to cite a retracted article, indicate the article’s retracted status in the References list and also include a citation and full reference for the retraction notice.

*RESPONSE: Thank you. This has been addressed.*

Reviewers' comments:

Reviewer #1: The article highlights an important association between family history of cancer and cancer attitude and beliefs. There are some aspects that authors may consider reviewing
Participants eligibility criteria is not defined well.

*RESPONSE: Data for the current study was collected as part of the CITIES project. The overall goal of the CITIES project was to define and describe the catchment area of the Ohio State University Comprehensive Cancer Center (OSUCCC). All Ohio residents between the ages of 21 to 74 were eligible for this study. The methods section has been updated to correct the age range. A variety of recruitment methods were used to gather a diverse sample. We briefly summarized the sampling methods in the manuscript. More detailed methods and sampling strategies have been described in the original CITIES manuscript and is cited in the methods:*

*Paskett ED, Young GS, Bernardo BM, Washington C, DeGraffinreid CR, Fisher JL, et al. The CITIES Project: Understanding the Health of Underrepresented Populations in Ohio. Cancer Epidemiol Biomarkers Prev. 2019;28(3):442-54.*

Under outcome variables why prostate screening is not mentioned.

*RESPONSE: Thank you for the thoughtful comment. Knowledge about prostate cancer screening was not included as a question in the CITIES survey; therefore, it could not be evaluated as an outcome in our study. Notably, prostate cancer screening guidelines are highly variable. Screening recommendations depend on an individual’s life expectancy and shared decision-making between patient and their provider.*

I don't see description for sample size calculation

*RESPONSE:* *A sample size calculation was not performed for this study. We considered if we would have an adequate sample size for analysis by considering the minimum counts needed per predictor. The maximum number of predictors used in the models was 9 variables, and with the minimum of 10 counts per predictor, the study is well over the sample of n=90 needed for analysis.*

2nd line in the discussion section on " We hypothesized that participants with a FDR with cancer would have more negative attitudes and beliefs about cancer" contradicts with line # 95-96 in introduction where FDR with cancer has been cited to related with more conscious attitude towards health screening.

*RESPONSE: The studies cited report a positive relationship between having a FDR with cancer and increased cancer screening behavior. It is thought that cancer attitudes and beliefs (CABs) may play a role in this association, but there are no studies to directly support this. Our study specifically examines the relationship between FDR and cancer attitudes and beliefs.*

Discussion
Overall length of this section seems very limited. Authors can consider adding more literature to compare and contrast the findings.

*RESPONSE: Thank you for your careful review and suggestion. The discussion section is updated with additional literature.*
