# Supplementary material for: The relationship between family history of cancer and cancer attitudes & beliefs within the Community Initiative Towards Improving Equity and Health Status (CITIES) cohort
Source: PLoS One. 2023 Jun 27;18(6):e0287629. doi: 10.1371/journal.pone.0287629 (PMC10298770; doi:10.1371/journal.pone.0287629)
Supplement: Attachment — Submitted filename: ResponsetoReviewers_CITIES.docx [file pone.0287629.s002.docx]

Thank you for your thoughtful review, comments, and suggestions. We believe we have now addressed these concerns and have detailed them below.

1. The justification provided is not entirely correct. While it is true that having a minimum count of 10 per predictor is a general rule of thumb, it is not the only consideration when determining sample size for a study. Other factors that should be taken into account include the effect size you want to detect, the desired level of statistical power, and the alpha level (level of significance) you plan to use. Without a formal sample size calculation, it is difficult to determine if the sample size is truly adequate for the study's research questions and hypotheses. Therefore, while the study might have enough data to conduct some form of analysis, the lack of a formal sample size calculation could be considered a limitation of the study.

*RESPONSE: We acknowledge that we did not have a sample size calculation for this study. However, the goal of the Community Initiative Towards Improving Equity and Health Status (CITIES) project was to develop core survey items and implement population surveys in the catchment area of the OSU Comprehensive Cancer Center. Since this was a one-time cross-sectional survey conducted between 2017 and 2018, and we are not quantifying any intervention effects or conducting comparisons to other populations, we do not think a post hoc sample size calculation is required. The current study is to describe cancer attitudes and beliefs among the study population within the OSUCCC catchment area. We included that the findings from this study is not generalizable to other populations as a limitation. We also described the differences of the study sample characteristics within the general Ohio residents. Therefore, we think we have addressed the limitation of the study design.*

2. Tabs. 3 & 4 results are not mentioned in the abstract and are not coherent with the main study objectives.

*RESPONSE: The manuscript has been revised to include the importance of tables 3 & 4.*

3. Need to improve the main focus of the manuscript with coherence in the objectives and results.

*RESPONSE: The manuscript has been revised to address coherence.*
